# Quantitative analysis of intrinsic and extrinsic factors in the aggregation mechanism of Alzheimer-associated Aβ-peptide

**DOI:** 10.1038/srep18728

**Published:** 2016-01-13

**Authors:** Georg Meisl, Xiaoting Yang, Birgitta Frohm, Tuomas P. J. Knowles, Sara Linse

**Affiliations:** 1 Department of Chemistry, University of Cambridge, Lensfield Rd, Cambridge, CB21EW, UK; 2 Department of Biochemistry and Structural Biology, Lund University, Lund, 22100, Sweden

## Abstract

Disease related mutations and environmental factors are key determinants of the aggregation mechanism of the amyloid-β peptide implicated in Alzheimer's disease. Here we present an approach to investigate these factors through acquisition of highly reproducible data and global kinetic analysis to determine the mechanistic influence of intrinsic and extrinsic factors on the Aβ aggregation network. This allows us to translate the shift in macroscopic aggregation behaviour into effects on the individual underlying microscopic steps. We apply this work-flow to the disease-associated Aβ42-A2V variant, and to a variation in pH as examples of an intrinsic and an extrinsic perturbation. In both cases, our data reveal a shift towards a mechanism in which a larger fraction of the reactive flux goes via a pathway that generates potentially toxic oligomeric species in a fibril-catalyzed reaction. This is in agreement with the finding that Aβ42-A2V leads to early-onset Alzheimer’s disease and enhances neurotoxicity.

Alzheimer’s disease is a devastating neurodegenerative disease. The amyloid beta peptide (Aβ) is implicated as a central factor in its etiology^1^,^2^,^3^; however, a molecular understanding of the connection is still largely lacking. Monomeric Aβ is unfolded, but the peptide has a high propensity to self-assemble into extended amyloid fibrils^3^. This aggregation process is associated with the formation of strongly neurotoxic species, including intermediate-sized aggregates, oligomers^4^,^5^, that seem to trigger neuronal death and whose formation can be catalysed by mature fibrils. To define the biological impact of protein aggregation, it is therefore crucial to understand the formation of both the final fibrillar aggregates and these oligomeric intermediates.

Protein aggregation has the characteristics of a nucleated growth process. Primary nucleation leads to the formation of the initial aggregates from soluble peptides, and addition of monomers in an elongation step underlies the further growth of such aggregates. It has become apparent, however, that primary nucleation is often a very slow process, and in many cases the majority of new aggregates are formed in autocatalytic secondary processes involving existing fibrils, for example through fragmentation of fibrils, or through interactions of monomers with fibrils in a process referred to as secondary nucleation. Such a double nucleation mechanism, including primary and secondary processes, describes quantitatively the aggregation data of the two major Aβ variants^6^,^7^,^8^. Secondary nucleation has been identified as an efficient generator of toxicity^6^.

Establishing the molecular mechanism of Aβ aggregation in terms of the underlying microscopic steps serves as a first important stage in the quest for a full understanding of the role of Aβ in Alzheimer’s disease. Finding the molecular driving forces for each step is a crucial next stage, which may provide a basis for finding means to interfere with the process and to redirect its flux towards the least toxic pathway^9^. A key question thus becomes the manner in which each of these steps are modulated by intrinsic (sequence) or extrinsic (solution conditions) factors, in particular those steps that give rise to toxicity.

*In vivo*, aggregation occurs in the presence of high concentrations of ions, small molecules and biopolymers such as proteins, nucleic acids, etc., as well as biological surfaces. Two approaches present themselves for dealing with this complexity: In a top-down approach, aggregation is studied in this highly complex environment where extrinsic or intrinsic factors are selectively removed or introduced to deduce their influence. In the bottom-up type approach we employ here, the aggregation mechanism is first studied in pure buffer and various extrinsic and intrinsic factors are introduced one by one to elucidate their effects. While the latter approach is a more direct route towards understanding the physico-chemical driving forces of each step in the aggregation mechanism, the former approach may reveale the importance of specific factors in the context of full biological complexity. Both approaches will need a detailed chemical kinetics methodology to further our understanding of Aβ self-assembly in a disease-relevant context.

Here we outline a general chemical kinetics approach to investigate factors that modulate the individual steps underlying the aggregation mechanism. We apply the approach to the A2V mutation associated with Alzheimer’s disease as an example of an intrinsic factor: The early-onset mutation, A2V, is reported to result in enhanced Aβ production and aggregation^10^,^11^ as well as changes in oligomer structure and hydrophobicity^12^. However, the aggregation mechanism of Aβ with the A2V mutation has not been studied.

We also study a variation in pH as an example of an extrinsic factor: The pH value varies slightly between body fluids and within cellular compartments, and even a relatively small change may serve as a mechanistic switch as recently found for the Parkinson’s disease-associated protein α-synuclein^13^.

## Results

### Experimental setup for reproducible data

Here the aggregation of Aβ was monitored as a function of time and peptide concentration using thioflavin T (ThT) as a reporter of fibril formation. The kinetic analysis of a system as complex as the reaction networks of protein aggregation requires global kinetic data of very high quality in order to yield robust and meaningful insights into the microscopic steps underlying the aggregation mechanism. To obtain such reproducible data of sufficient quality, the reaction conditions need to be precisely controlled and specified at all levels. In this context the following eleven points were given careful consideration:

#### 1. Homogeneous sequence

Even a small amount of peptide with a minor variation in sequence can lead to retardation of aggregation^14^. Hence mixtures of peptides with differing sequences are not suitable for kinetic analysis. For a 42-residue peptide, cloning and recombinant peptide expression is therefore required. The high fidelity of the ribosome provides samples with extremely high sequence homogeneity. Peptide synthesis by contrast suffers from a higher error rate, yielding samples with a large number of similar sequences, which makes it difficult, if not impossible, to isolate a unique peptide during purification and results in significant batch-to-batch variation^14^.

#### 2. Sufficient amounts of peptide

The need for multiple monomer concentration (see below) means that an efficient expression system is needed. Tag-free expression eliminates the need for cleavage and post-cleavage purification. The Aβ42 peptides are thus expressed “as is” in *Escherichia coli*. The formation of inclusion bodies, favoured by the high aggregation propensity, protects Aβ against proteolytic degradation, a common problem during expression of soluble unstructured peptides. Formation of inclusion bodies moreover allows for high expression levels of the toxic peptides.

#### 3. Pure peptide

**Figure 1:**
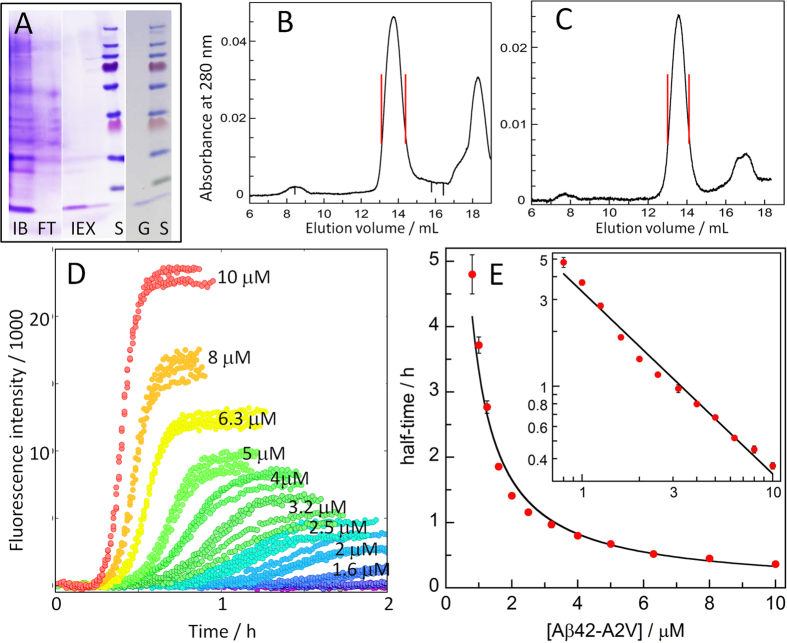
Aβ42-A2V purification, monomer isolation and aggregation kinetics experiments. (**A**) SDS PAGE (10–20% Tris-Tricine gel) of urea-dissolved inclusion bodies (IB), flow-through of IEX resin (FT), eluted fractions from ion exchange (IEX), pooled sample after gel filtration (G), and Mw standards (S) with the lowest marker at 10 kDa (the right-most standard lane contains a spill-over of Aβ seen below the 10 kDa marker). (**B**) Gel filtration on a Superdex75 1 × 30 cm column of a lyophilized aliquot from the pool (G) in panel A after dissolution in 6 M GuHCl. The collected fraction was between the red marks. (**C**) Monomer isolation by gel filtration on a Superdex75 1 × 30 cm column of the lyophylized monomer fraction from panel B after dissolution in 6 M GuHCl. The collected fraction between the red marks was used in the aggregation kinetics experiments. (**D**) Examples of aggregation kinetic data, four technical replicates of ThT fluorescence as a function of time, with initial monomer concentration between 0.8 and 10 μM as indicated next to the respective curves from 1.6 μM and up. (**E**) Half time of the aggregation process as a function of initial monomer concentration. The solid line is the best fit of a power function (A*m^γ^), the exponent γ of the best fit is −1.00. The inset shows the same data on a logarithmic scale to illustrate the curvature and deviation from a straight line.

The high sensitivity of the aggregation reaction to the solution conditions means that to obtain reproducible data and mechanistic insights, all forms of impurities need to be removed, to at least the level where they cannot be detected by NMR spectroscopy (small molecules) or on silver-stained gels (proteins). A robust purification protocol is needed, and for the aggregation prone and surface active Aβ peptide, losses on chromatographic resins need to be limited by using a small number of highly efficient steps. The formation of inclusion bodies facilitates the rapid removal of *E. coli* proteins by repeated sonication and centrifugation at the initial stage of purification, therefore metal ion chelation by EDTA is the only protease inhibition needed. The subsequent ion exchange purification is performed in batch mode to avoid concentrating the peptide on the resin. This is followed by size exclusion purification^15^ using spin filters or chromatography (Fig. 1A), and additional rounds of size exclusion chromatography are crucial to ensure the removal of traces of small molecules and proteins from *E. coli* (Fig. 1B,C).

#### 4. Well-defined initial state

At the initial stages of a mechanistic [Fig f2][Fig f3]investigation, reactions starting from pure monomer represent the most well-defined starting condition. Due to the positive feedback mechanisms governing the aggregation of Aβ^6^,^7^,^8^, low concentrations (sub nano-molar) of preformed aggregates can have a significant effect on the kinetics of the reaction (see for example Fig. 4E and 5E below). Therefore, when the aim is to study non-seeded aggregation starting from pure monomer, all preformed fibrillar or oligomeric species need to be removed by gel filtration immediately prior to starting the kinetic experiment. When the aim is to study seeded aggregation, preformed seeds are collected when the final plateau in the ThT trace is reached. These seeds are added in a controlled manner at defined concentrations to freshly isolated monomer.

#### 5. Low-binding surfaces

The aggregation process can be strongly influenced by surfaces that adsorb Aβ and may catalyse or inhibit nucleation, leading to either acceleration or retardation^16^,^17^ depending on the affinity for the surface^18^. Inert surfaces are therefore preferred. In a study using a range of materials, PEGylated surfaces were the only surfaces for which no binding of Aβ could be detected^19^. In the current study PEGylated multi-well plates are used to minimise Aβ adsorption to materials surfaces.

#### 6. Minimized and controlled air-water interface

Aβ has a tendency to partition to the air-water interface, where heterogeneous nucleation may occur, as recently shown for a-synuclein^20^. The air-water interface is minimised through extensive degassing of all buffers and using low-area multi-well plates^21^.

#### 7. Defined solution conditions

The high sensitivity of the process to the solution conditions requires full control over buffer composition, pH, ionic strength, temperature, etc. To monitor the effect of a specific factor, all other conditions need to be kept constant.

#### 8. Absence of co-solvents

Co-solvents, e.g. DMSO and HFIP, may affect the aggregation process *per se* and may also alter the magnitude of the effect of any studied factor. Therefore, no co-solvents are added and gel filtration is used for monomer isolation.

#### 9. Faithful representation of the aggregate fibril mass by the measurement technique

The concentration of the reporter dye, ThT, is optimised through a stringent set of controls. On the one hand it is crucial to ensure that the presence of ThT does not affect the kinetics of amyloid formation^7^,^22^. On the other hand it is necessary to verify that the signal, which is an indirect measure of fibril concentration, is directly proportional to the mass concentration of amyloid fibrils^6^. At excessive ThT concentrations, the fluorescence of fibril-bound ThT is quenched by excess ThT (micelles), while an insufficient ThT concentration may deteriorate the kinetic data at the end of the reaction.

#### 10. Monomer concentration range and spacing

To provide sufficient constraints in the fitting of models, multiple samples with monomer concentrations varying over at least one order of magnitude are essential (Fig. 1D). Logarithmic spacing of peptide concentrations (e.g. each concentration is 20% lower than the one above) provides the most stringent discrimination between models.

#### 11. Quiescent conditions

Agitation of samples, sometimes at very high shaking speed, has been used in the past to increase reproducibility in cases where nucleation is compromised by less stringent control of factors 1–9. However, agitation is not required to obtain reproducible data^6^. In fact, quiescent conditions will provide more relevant mechanistic insights and are therefore much preferable. Since fibrils grow from their ends, the aggregation process is accelerated by agitation which leads to increased fragmentation of fibrils and thereby to production of more ends for elongation. A kinetic analysis of data obtained for agitated samples typically finds a fragmentation-dominated mechanism^23^. By contrast, the use of data obtained under quiescent conditions may provide insights into microscopic processes that are more likely to occur under *in vivo* conditions.

### Kinetic analysis

**Figure 2 f2:**
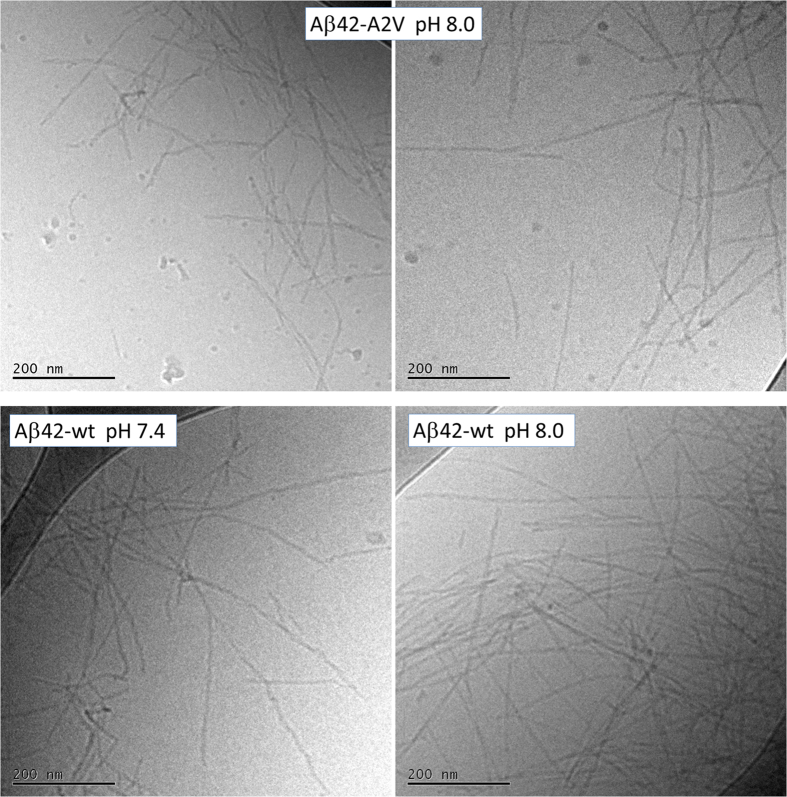
Cryo-TEM images of Aβ42-A2V fibrils formed at pH 8.0 (top two fields) and Aβ42-wt fibrils (bottom two fields formed at pH 7.4, left, and pH 8.0, right). The fibrils were collected after reaching the final plateau in the ThT fluorescence curve. The scale bar is 200 nm.

The starting point for the kinetic analysis are the time-dependent data for fibril formation as monitored in non-binding plates under quiescent conditions for initially monomeric solutions at a range of peptide concentrations (Figs 1D, 4A,B and 5A,B). Fibril formation is verified using electron microscopy, which reveals similar fibrils for Aβ42-A2V and Aβ42 wt at both pH values (Fig. 2).

The kinetic analysis commences with the determination of the half time, the time at which half the protein has converted into its aggregated form. Its dependence on the initial monomer concentration, *m*, can be modelled as a power function (A* m^*γ*^ with scaling exponent γ and constant *A*, Fig. 1D); the scaling exponent serves as a rough guide to possible models^24^,^25^.

**Figure 3 f3:**
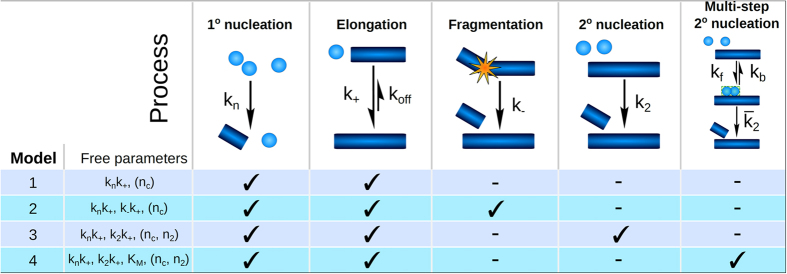
Mechanisms of aggregation. Schemes of the microscopic steps that make up the reaction network in the four different models (model 1, 2, 3 and 4) tested in the kinetic analyses. The free fitting parameters for each model are given as well, the reaction orders are given in brackets, as they are either fitted (in models 1b, 2b, 3b and 4b) or fixed to their value found in the case of wt Aβ42 at pH8 (in models 1a, 2a, 3a and 4a). Further details given in Fig S3.

**Figure 4:**
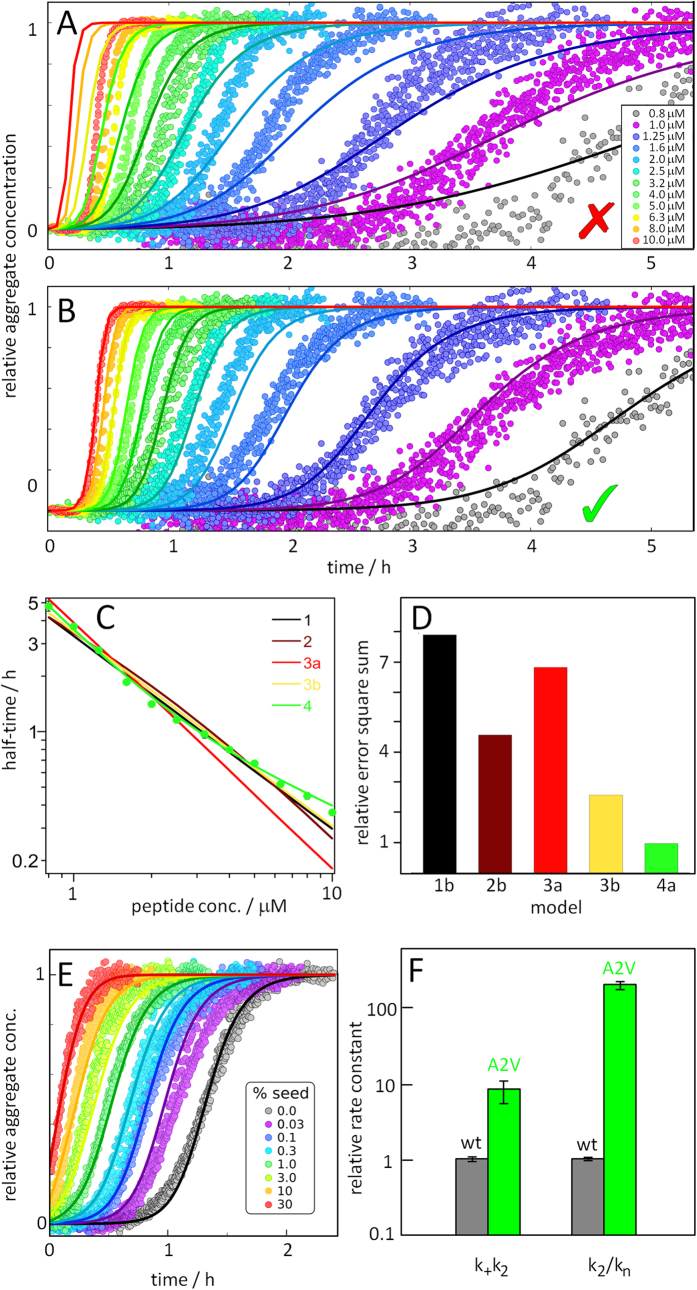
Kinetic analysis of Aβ42-A2V. (**A**,**B**) Normalized time courses (ThT fluorescence data points) for aggregation reactions starting from 0.8 (grey) to 10 μM (red) Aβ42-A2V monomer in 20 mM NaP, 0.2 mM EDTA, 0.02% NaN_3_, pH 8.0. Each colour shows four technical replicates at each concentration. The solid lines represents the best fits of model 3a (A) and model 4a (B) and plots for all models are shown in Figure S1. (**A**) The best fit with a model consistent with the Aβ42 wt data: primary nucleation, secondary nucleation and elongation with fixed reaction orders n_c_ = n_2_ = 2 (as for wt) (model 3a) and (**B**) the best fit with a model including primary nucleation and multi-step secondary nucleation (model 4a). (**C**) The half time as a function of peptide concentration from the best fit of each model is compared to the experimental data. (**D**) Error square sum, a measure of the goodness of the fit, of each model relative to model 4a. The models, with the number of fitting parameters given in brackets, are: **Model1b** Primary nucleation and elongation (2). **Model2b** Primary nucleation, fragmentation and elongation (3). **Model3a** Primary nucleation, secondary nucleation and elongation (2). **Model3b** Primary nucleation, secondary nucleation and elongation (4). **Model4a** Primary nucleation, multi-step secondary nucleation and elongation (3). Although involving one less free parameter than model 3b, model 4a yields a lower error. See Figure 3 for the processes and parameters for each model. (**E**) Normalized aggregation kinetics data for samples that initially contain 2.3 μM monomer supplemented with 0.03, 0.1, 0.3, 1, 3, 10 or 30% seeds (in monomer equivalents) confirm the strong role of surface-catalyzed secondary nucleation. The solid lines are fits of model 4a, using the parameter values found above and one free parameter, the elongation rate constant k_+_. (**F**) Comparison of k_2_k_+_and k_2_/kn for Aβ42-A2V relative to Aβ42-wt. In particular note the large increase in the relative importance of secondary over primary nucleation.

**Figure 5:**
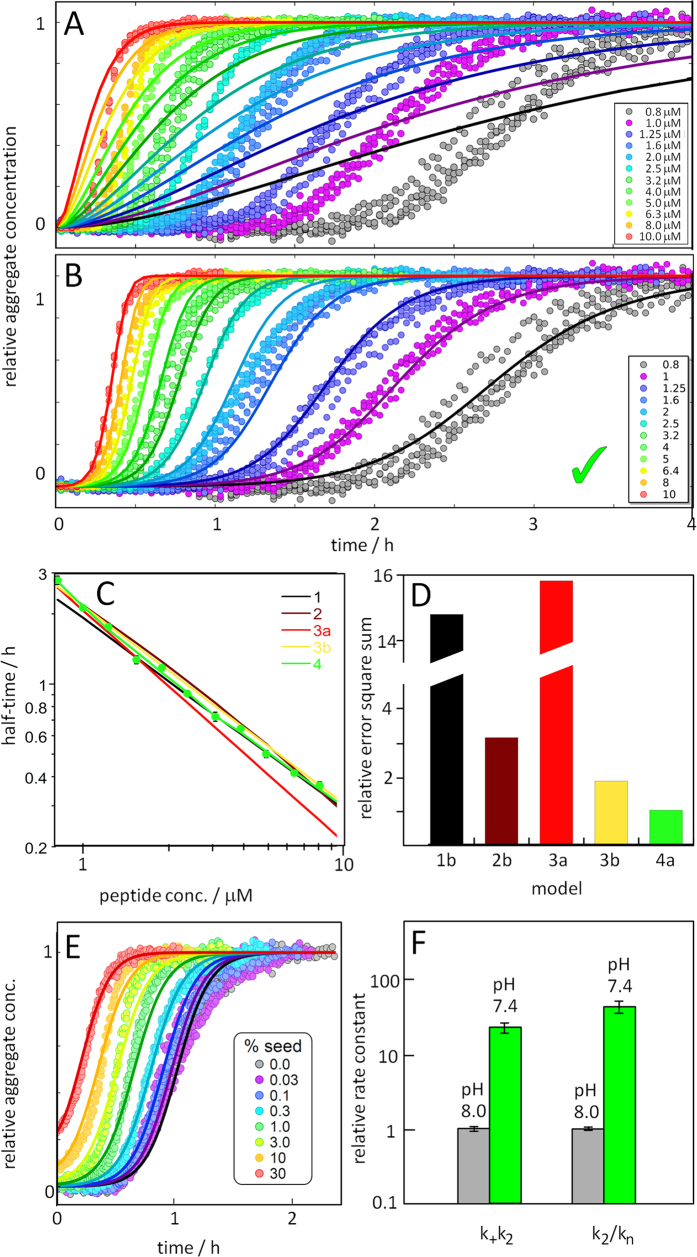
Kinetic analysis of Aβ42 wt at pH 7.4. (**A**) Normalized time courses (ThT fluorescence) for aggregation reactions starting from 0.8 (grey) to 10 μM (red) Aβ42 monomer in 20 mM NaP, 0.2 mM EDTA, 0.02% NaN_3_, pH 7.4. Each colour shows four technical replicates at each concentration. The solid lines represents the best fits of model 3a (A) and model 4a (B) and plots for all models are shown in Figure S2. (**A**) The best fit with a model consistent with the Aβ42 wt data at pH 8.0: primary nucleation, secondary nucleation and elongation with fixed reaction orders of n_c_ = n_2_ = 2 (model 3a). (**B**) The best fit with a model including primary nucleation and multi-step secondary nucleation (model 4a). (**C**) The half time as a function of peptide concentration from the best fit of each model is compared to the experimental data. (**D**) Error square sum, a measure of the goodness of the fit, of each model relative to model 4a. The models, with the number of fitting parameters given in brackets, are: **Model1b)** Primary nucleation and elongation (2). **Model2b** Primary nucleation, fragmentation and elongation (3). **Model3a** Primary nucleation, secondary nucleation and elongation (2). **Model3b** Primary nucleation, secondary nucleation and elongation (4). **Model4a** Primary nucleation, multi-step secondary nucleation and elongation (3). Although involving one less free parameter than model 3b, model 4a yields a lower error. See Fig. 3 for the processes and parameters for each model. (**E**) Normalized aggregation kinetics data for samples that initially contain 2.2 μM monomer supplemented with 0.03, 0.1, 0.3, 1, 3, 10 or 30% seeds (in monomer equivalents) confirm the strong role of surface-catalyzed secondary nucleation. The solid lines are fits of model 4a, using the parameter values found above and one free parameter, the elongation rate constant k_+_. (**F**) Comparison of k_2_k_+_ and k_2_/kn for Aβ42-wt at pH 7.4 relative to pH 8.0.

Different kinetic models (Fig. 3) are fitted globally to the kinetic data as a function of peptide concentration and time (Figs 4A,B and 5A,B, S1, S2). This yields a minimal combination of microscopic steps and the associated rate constants compatible with all data. The models considered here are based on a nucleated linear polymerisation model, including secondary pathways in which existing aggregates increase the rate of formation of new aggregates either through fragmentation or through surface catalysed secondary nucleation. Each model is globally fitted to the data, fixing the reaction orders for primary nucleation (n_c_) and for secondary nucleation (n_2_) to the values found for Aβ42 (n_c_ = n_2_ = 2)^6^. These fits are shown in the left column of Figures S1 and S2. Each model is also globally fitted to the data using variable reaction orders, as shown in the right column of Figures S1 and S2.

### Intrinsic factors – the A2V mutation

Examples of unseeded aggregation kinetics data for A2V are shown in Figs 1D and 4A,B, and the extracted half-times are shown in Figs 1E and 4C. The average scaling exponent, *γ,* is found to be −1.00. This value is consistent both with a primary dominated and with a secondary nucleation dominated mechanism and allows us to discard a fragmentation dominated mechanism, which would result in γ ∼ −0.5. Whilst the average scaling *γ* already provides some constraints on possible mechanisms, we also find that *γ* in fact varies with the monomer concentration, reflected by the slight curvature in a double logarithmic plot of half time versus monomer concentration: at low monomer concentrations the aggregation is strongly monomer-dependent, leading to a strong scaling of *γ* ∼ −1.5; at high monomer concentrations the aggregation becomes less dependent on monomer concentration leading to a scaling of *γ* ∼ −0.5. This variation of *γ* with the monomer concentration is indicative of a process that changes its monomer dependence as a function of monomer concentration, such as a saturating process. Any proposed mechanism will have to satisfy all these constraints, already greatly reducing the number of models that need to be considered in the fitting analysis.

To illustrate the power of the global fitting process, we also include the fits of a simple nucleated polymerisation model, without any secondary pathways, and then gradually increase the complexity. The models and the processes involved, are schematically depicted in Fig. 3. The fits to all models are shown in Figure S1, and the best fit in Fig. 4B.

Model 1 includes primary nucleation and elongation only. It can reproduce neither the dependence of the half time on the peptide concentration nor the steepness in the transition region of individual aggregation curves observed for Aβ42-A2V. This is as expected, as the sharp increase after a long lag-time observed in our data is characteristic of the presence of secondary processes.

Model 2 includes fragmentation in addition to primary nucleation and elongation. Although the individual kinetic curves now better match the data in terms of their shape, their dependence on monomer concentration can still not be reproduced by this model^6^, as expected from the half time analysis. This emphasizes how crucial a global fit of multiple monomer concentrations is: a single concentration alone does not provide sufficient constraints and, for example, would not have allowed us to discard a fragmentation dominated model in this case.

Model 3 does not include fragmentation, but instead a monomer-dependent secondary nucleation mechanism. The curve shape is similar to Model 2 which gives a sharp increase after a long lag time. However, because the secondary nucleation process depends on the concentration of free monomers, the curves at different monomer concentrations will be more spread out compared to the fragmentation model. This is reflected by the scaling exponent *γ* which is approximately −0.5 in the case of fragmentation (i.e. a weak dependence on monomer), but can be much more negative in the case of secondary nucleation (i.e. a stronger monomer dependence such as e.g. *γ* = *−*1.33 in the case of wt Aβ42). This model describes the aggregation of wt Aβ42 and the fits to the current dataset represent a significant improvement over model 2, when setting the reaction orders of primary and secondary nucleation to be the same as in the wild type (model 3a). When the reaction orders of both primary and secondary nucleation are allowed to vary from their wt values (Model 3b), the fit is found to improve further. However, neither fit manages to capture the data both at low and at high monomer concentrations simultaneously.

Model 4 is an extension of model 3, in which the secondary nucleation process is treated in more detail: we consider it explicitly as a 2-step mechanism with a monomer-dependent initial attachment step, followed by a monomer-independent rearrangement/detachment step, a mechanism that was previously found to describe the aggregation Aβ40^7^. This 2-step model naturally produces Michaelis-Menten-like saturation kinetics, yielding overall a monomer-dependent process in the limit of low monomer concentrations and a saturated, monomer-independent process at high monomer concentrations. The 2-step secondary nucleation model is finally able to account for the variation in scaling exponent that was already observed in the initial half time analysis and the change in slope in the t_1/2_ plot is reproduced (Fig. 4C). Incorporation of saturation of the secondary nucleation process also leads to improved global fits. The model is now able reproduce curve shapes as well as the monomer dependence over the entire range of monomer concentrations, to within experimental error. Thus, Aβ42-A2V is fitted by model 4, which includes primary nucleation, multi-step secondary nucleation and elongation and the parameter values are k_+_k_n_ = 38 ± 8 M^−2^s^−2^, k_+_k_2_ = 3.4.10^11^ ± 2.4.10^10^  M^−3^s^−2^, √K_M_ = 2.6 ± 0.5 μM, fixed n_c_ = n_2_ = 2.

### Extrinsic factors – pH 7.4 vs pH 8.0

The data for Aβ42 wt at pH 7.4 were analyzed in the same manner as the data for the aggregation of A2V above (Figs 5 and S2). The average scaling exponent γ is found to be −0.9, which again allows us to discard a fragmentation dominated mechanism. The fits of all models are shown in Figure S2, and models 3 and 4 in Fig. 5A,B. Model 4 is able to reproduce both the curve shapes and the monomer dependence over the entire range studied without adjustment of the reaction orders from the values found at pH 8 (Fig. 5B). However, model 3a, which fits the aggregation data of Aβ42 wt at pH 8.0^6^, performs poorly for the pH 7.4 data (Fig. 5A,D). Thus, Aβ42 wt at pH 7.4 is fitted by model 4, which includes primary nucleation, multi-step secondary nucleation and elongation and the parameter values are k_+_k_n_ = 480 ± 60 M^−2^s^−2^, k_+_k_2_ = 9.0.10^11^ ± 1.4.10^11^ M^−3^s^−2^, √K_M_ = 1.1 ± 0.4 μM, with fixed n_c_ = n_2_ = 2.

### Prediction and Verification using seeding

In order to verify predictions of the chosen model or to extend the descriptions of the aggregation from pure monomeric protein, additional components can be added to increase the complexity of the system. In this context, one particularly useful system is the aggregation of monomeric samples, supplemented at time zero with preformed seed fibrils at low concentrations (Figs 4E and 5E). First, this constitutes an independent test for the presence of secondary processes: the direct elongation of a low concentration of seeds (usually nM) is a negligible contribution to the overall fibril mass. However, if secondary processes are present, the lag-time is dramatically shortened due to the positive feedback resulting from seeds bypassing primary nucleation and catalysing the production of more seeds via the secondary process. Second, the kinetics of seeded experiments can be predicted from the fits to the data for un-seeded samples, thereby representing a strong test of the choice of model. Third, seeded experiments, in combination with a measurement of the average seed size (see Methods), allow one to estimate the rate constants of elongation, k_+_, whereas only the combined rate constants k_+_k_n_ and k_+_k_2_ can be obtained from the data for the unseeded reactions (see SI).

For Aβ42 at pH 7.4 we observe a significant reduction in lag-time upon addition of small amounts of seeds, similar to Aβ42 at pH 8.0, confirming the importance of a secondary process as the dominant mechanism of fibril multiplication. Using the rates obtained in the unseeded case, the seeded data was reproduced to well with only a single free fitting parameter (Fig. 5E).

For A2V we observe an even more significant reduction in lag-time. It is reduced by 25% already at seed concentrations as low as 0.7 nM, confirming an even higher dominance of secondary nucleation. The observed seeding effect for Aβ42-A2V is considerably stronger than that reported previously for Aβ42 wt, a system for which the lag phase is significantly shortened with 1% w/w seed^6^,^8^,^9^. In the case of A2V, the lag phase is essentially absent already at 1% w/w seed (Fig. 4E). Moreover, fits of the seeded data, with all rate constants constrained to the values determined from fits of the un-seeded data, agree well with experiment, further strengthening our choice of model.

## Discussion

The results of the kinetic analysis imply that the A2V mutation causes a shift in the reactive flux towards higher dominance of a secondary pathway that generates oligomers through nucleation of monomers on the fibril surface. Comparing the rate constants obtained here to the rate constants of Aβ42^6^, we find that the combined rate constant of elongation and secondary nucleation, k_+_k_2_, is an order of magnitude higher for Aβ42-A2V compared to Aβ42 wt (Fig. 4F).

For Aβ42 wt, the majority of new aggregates are created through secondary rather than primary nucleation^6^,^8^,^9^, and this feature is significantly enhanced for Aβ42-A2V; we find that the relative importance of secondary nucleation over primary nucleation has increased by two orders of magnitude (Fig. 4F).

The A2V mutation was recently found among patients suffering from early onset Alzheimer’s disease^10^. The Aβ42-A2V variant is found to be more toxic compared to Aβ42 wt in cell cultures^10^ and in a *Caenorhabditis elegans* model^11^, and neuropathological investigations have revealed larger Aβ deposits in the brain of A2V carriers^26^. Previous studies of Aβ42 wt have shown that the pathway involving secondary nucleation is the main route to generation of toxicity in cell cultures^6^, and the increased toxicity caused by the A2V substitution^10^, ^11^, ^12^ correlates with the enhancement of this pathway found here. In line with these findings, the observed seeding effect, which is directly related to the efficiency of the secondary pathway, is considerably stronger for A2V than for wt Aβ42 (Fig. 4E). To illustrate this difference, we calculate the critical seed concentration, C_s_. When the concentration of aggregates is higher than this critical value, secondary nucleation produces more nuclei per second than primary nucleation. In this case the critical concentration is simply given by C_s_ = k_n_/k_2_. For Aβ42-A2V we obtain C_s_ = 0.1 nM, and for Aβ42 wt C_s_ is estimated to 20 nM^6^.

Very interestingly, both the intrinsic and the extrinsic variation studied here lead to the same qualitative change of the mechanism from Aβ42 wt at pH 8.0. We find that in both modified systems there is an increased dominance of secondary nucleation over primary nucleation and a saturation of secondary nucleation, which is very likely due to a saturation of the fibril surface in bound species, whereas no saturation is observed at the same concentrations in Aβ42 wt at pH 8. The strong increase in secondary nucleation rate found for both factors may originate in an increased affinity of monomers for the fibril surface. The A2V substitution leads to an increase in hydrophobicity by 1 kJ/mol^27^,^28^,^29^ for each monomer and per monomer unit in fibrils. This will likely increase the affinity between monomers and fibrils as well as between monomers. The significant effect of the A2V substitution on secondary nucleation thus suggests that this process is strongly driven by the hydrophobic effect. The pH switch, on the other hand, modulates electrostatic interactions as it leads to a change in peptide net charge towards a less negative value, from between −4 and −3 at pH 8.0 to closer to −3 at pH 7.4. In this case, increased affinity comes from attenuation of the electrostatic repulsion between monomer and between monomers and fibrils.

We show here that to deduce a mechanistic change in terms of which microscopic steps are enhanced, it is crucial to use global fitting of data at multiple monomer concentrations^24^. In a previous study, the aggregation kinetics of synthetic Aβ40-A2V was compared to Aβ40 wt at one peptide concentration, leading to the conclusion that the overall aggregation rate is increased by the mutation^10^. A single kinetic trace can be fitted by several models, but when multiple concentrations are used, most models fail to reproduce both the individual curve shapes and the dependence on monomer concentration. Moreover, the sharp increase of ThT fluorescence intensity at the end of an initially flat lag phase, observed here at every monomer concentration of Aβ42-A2V, cannot be captured by model 1, which includes only primary nucleation and elongation. Such curve features are characteristic of mechanisms that include also secondary processes. The positive feedback due to fibrils accelerating the formation of more fibrils, results in a run-away process and a sudden increase in aggregate mass, which is believed to be one of the origins of the difficulty in curtailing amyloid diseases.

## Concluding remark

The global kinetic analysis of aggregation data represents a powerful tool in extracting mechanistic information and insights into the complex reaction networks of aggregation. However, high quality experimental data obtained at several peptide concentrations under careful control of the experimental conditions and a knowledge of all factors and conditions in the aggregation reaction are required. In the framework presented here, we have shown how these challenges can be addressed to obtain quantitative and fully reproducible data of high quality. Within this framework, we are able to study the effect of various intrinsic and extrinsic factors on the aggregation of Aβ42. Here we investigated an extrinsic factor (the variation of pH) and an intrinsic factor (the A2V mutant). Through careful analysis we find that these seemingly different factors lead to similar mechanistic changes; the rate and dominance of secondary nucleation is enhanced both by the A2V substitution and by a small decrease in pH, suggesting a common origin of these two effects, namely an increased binding affinity of monomers for the catalytic fibril surface. In terms of molecular driving forces, this likely originates in the increased hydrophobicity (A2V) or the reduced electrostatic repulsion (pH reduction). Both of these effects lead to an increased rate of generation of oligomers, which in the case of Aβ42-A2V correlates with its increased neurotoxicity.

More generally, our approach for the systematic study of the aggregation of Aβ and its sequence variants, under various solution conditions provides a solid footing for studies of the similarities and differences in aggregation mechanism. In addition to providing mechanisms for separate systems, a consideration of these effects in relation to each other can yield a deeper understanding of the underlying determinants that govern the rates of the individual processes by comparing the parallels and differences in the mechanistic effect of different factors. The approach may easily be extended to other disease related mutants and environmental factors to build up a comprehensive picture of how intrinsic and extrinsic factors alter the aggregation kinetics. A better understanding of these effects on each of the microscopic processes may provide the means to interfere with distinct steps in the aggregation mechanism.

## Methods

### Cloning and expression of Aβ peptides

A synthetic gene with *E. coli* optimised codons for Aβ(M1-42) with the A2V mutation cloned into the Pet3a vector was purchased from Genscript (Piscataway; New Jersey, USA). Aβ(M1-42) wt and A2V peptides, here called Aβ42 wt and Aβ42-A2V, were expressed in *E. coli* strain BL21 DE3 PLyS star (Invitrogen) in LB medium with 50 mg/l ampicillin and 30 mg/l chloramphenicol. Well-isolated small colonies from freshly transformed vector were used to innoculate 50 mL overnight cultures grown in 250 mL baffled flaks at 37 °C with 125 rpm shaking. These were diluted 1:100 into 500 mL day cultures grown in 2500 mL baffled flaks at 37 °C with 125 rpm shaking. 0.4 mM IPTG was added at OD 0.6–1.0 and the cells were harvested after another 3.5–4 h by centrifugation for 10 minutes at 6000 g.

### Purification of the Aβ42 wt peptide

Aβ42 wt was purified as described^15^ except that two rounds of gel filtration replaced the size exclusion filters.

### Purification of the Aβ42-A2V peptide

Cell pellet from 2.0 L culture was sonicated in 40 mL 10 mM Tris/HCl, 1 mM EDTA, pH 8.5 (Buffer A) using a sonicator tip (half horne, 50% duty cycle, maximum output, 30–90 s). This step was performed in a glass beaker surrounded by an ice-water slurry. Inclusion bodies were isolated by centrifugation at 4 °C, 15000 g for 10 min. Two more rounds of sonication and centrifugation were performed. The inclusion bodies were dissolved in 40 mL 8 M urea in buffer A (IB in Fig. 1A), and diluted 4-fold in buffer A. 20 mL DEAE cellulose (Whatman DE23, equilibrated in buffer A with 2 M urea) was added and the slurry was left on ice for 30 min (with stirring now and then using a glass rod). The following anion exchange purification step was performed in batch mode to avoid concentration of the peptide on the resin. The solution (FT in Fig. 1A of the main manuscript) was removed and the resin was washed with buffer A with 2 M urea in a Büchner funnel on a vacuum flask. The following washes were performed in sequence in buffer A: 2 × 20 mL 1.8 M urea + 10 mM NaCl, 2 × 20 mL 1.6 M urea + 10 mM NaCl, 2 × 20 mL of 1.4 M urea + 25 mM NaCl, 2 × 20 mL of 1.2 M urea + 25 mM NaCl. The peptide was then eluted in 3 × 20 mL buffer A with 1 M urea + 125 mM NaCl (IEX in Fig. 1A), followed by 2 × 20 mL buffer A with 1 M urea + 200 mM NaCl. The resin was stirred using a glass rod and incubated in each wash or eluent solution in the funnel for up to five minutes followed by removal of the liquid using the vacuum flask. The use of successively reduced urea concentration during washing and elution increases the yield of aggregation-prone Aβ42 variants. The eluted fractions were examined using agarose gel electrophoresis and SDS PAGE. Fractions dominated by Aβ42 monomer were isolated from remaining *E. coli* proteins, aggregates and small molecule contaminants using size exclusion chromatography on a G50 superfine column at 4 °C. The eluted fractions were examined using UV absorbance, agarose gel electrophoresis and SDS PAGE (G in Fig. 1A).

### Chemicals

All chemical were of analytical grade. All buffers were extensively degassed.

### Preparation of samples for kinetics experiments

Aliquots of purified Aβ42 wt or Aβ42-A2V were dissolved in 6 M GuHCl, and monomer isolated by gel filtration on a Superdex 75 column in 20 mM sodium phosphate buffer, pH 8, with 200 μM EDTA and 0.02% NaN_3_ (mutants) or 20 mM Hepes/NaOH, pH 8.0 (wild-type). This step is absolute key to reproducible kinetics and has to be performed. The center of the monomer peak was collected on ice and lyophilized. The sample was again dissolved in 6 M GuHCl, and monomer isolated by gel filtration on a Superdex 75 column in 20 mM sodium phosphate buffer, pH 8, with 200 μM EDTA and 0.02% NaN_3_ (mutants) or 20 mM Hepes/NaOH, pH 8.0 (wild-type). The gel filtration steps remove traces of pre-existent aggregates and exchanges the buffer to the one used in the fibril formation experiments. The peptide concentration was determined from the absorbance of the integrated peak area using ε_280_ = 1400 l mol^−1^cm^−1^ as calibrated using quantitative amino acid analysis. The monomer generated in this way was diluted with buffer to 8 μM and supplemented with 6 μM thioflavinT (ThT, Calbiochem) from a 1.2 mM stock (in water, filtrated through 0.2 μm filter). Samples for the kinetic experiments were set up with logarithmic spacing of peptide concentrations (with each concentration 20% lower than the one above); this provides the most stringent distinction between models because chemical reactions are governed by activities (proportional to the logarithm of the concentration). The isolated monomer is kept in low-binding tubes close to 0 °C during the time it takes to prepare dilution series or other sample variations. Each sample was then pipetted into four wells of a 96 well half-area plate of black polystyrene with a clear bottom and PEG coating (Corning 3881, Massachusetts, USA), 100 μL per well. The whole setup was repeated twice for each mutant. The ThT concentration is optimised^6^ to the one giving the highest signal for a given fibril concentration. Lower concentration means insufficient ThT concentration to give reliable shape of the curves at the end of the reaction, and higher concentrations will suffer from quenching of the fluorescence due to micelle formation of excess free ThT.

### Kinetic assays

Assays were initiated by placing the 96-well plate at 37 °C under quiescent conditions in a plate reader (Fluostar Omega or Fluostar Optima BMGLabtech, Offenburg, Germany). The ThT fluorescence was measured through the bottom of the plate every 60 s with a 440 nm excitation filter and a 480 nm emission filter.

### Kinetic assays with preformed fibrils

The seed fibrils were collected directly after reaching the final plateau in ThT fluorescence and immediately added at several dilutions to 4 μM fresh monomer in 20 mM sodium phosphate buffer, pH 8, with 200 μM EDTA, 6 μM ThT and 0.02% NaN_3_. The ThT fluorescence was monitored for 1.5 hours to verify the formation of fibrils. The samples were then collected from the wells into low-bind tubes (Axygen Scientific, Union City, California, USA). For the controlled experiments with different seed concentrations, the fibril concentration in each sample was then calculated from the dilution factor relying on very slow dissociation of monomers from fibrils, meaning that the fibril concentration does not decay during the short lag time of the seeded experiment. A series of samples were prepared with twice the desired final fibril concentration, and 50 μl placed in each well. Fresh monomer was isolated by gel filtration as above and diluted to 8 μM in 20 mM sodium phosphate, pH 8, containing 200 μM EDTA, 6 μM ThT, 0.02% NaN_3_, and 50 μl loaded to each well using a multi-channel pipette after which the plate was placed in the plate reader and the ThT fluorescence monitored every 60 s under quiescent conditions at 37 °C.

### Cryogenic Transmission Electron Microscopy (*cryo*-TEM)

The samples used for *cryo*-TEM were prepared and incubated in the same way as for the kinetic aggregation studies. Samples with 10 μM of Aβ42-A2V or Aβ42-wt were monitored by ThT and collected at the plateau (both first and second plateau for the Aβ42:Aβ40 mixture) and kept at 4 °C (maximum time kept at 4 °C was over night) until imaged by cryo-TEM. Specimens for electron microscopy were prepared in a controlled environment vitrification system (CEVS) to ensure stable temperature and to avoid loss of solution during sample preparation. The specimens were prepared as thin liquid films, < 300 nm thick, on lacey carbon filmed copper grids and plunged into liquid ethane at −180 °C. This leads to vitrified specimens, avoiding component segmentation and rearrangement, and water crystallization, thereby preserving original microstructures. The vitrified specimens were stored under liquid nitrogen until measured. An Oxford CT3500 cryoholder and its workstation were used to transfer the specimen into the electron microscope (Philips CM120 BioTWIN Cryo) equipped with a post-column energy filter (Gatan GIF100). The acceleration voltage was 120 kV. The images were recorded digitally with a CCD camera under low electron dose conditions.

### Fibril length estimate

The average fibril length was estimated from the cryo-TEM image, by measuring the end to end distance of 25 individual fibrils and the thickness of 32 fibrils. The average length of the seed fibrils was found to be 400 (+−200) nm with a cross sectional area of 100 ( + −40) nm^2^. Assuming a density of 1.3 g/ml seeds hence consist of ~7000 monomers, accurate to within a factor of 3. This value can be used as the global constant L(0) = M(0)/P(0), the ratio of initial fibril mass over initial fibril number, in the fits of seeded data to obtain a rough estimate of the elongation rate constant.

### Kinetic analyses

The kinetic modelling of the data closely follows the approach presented in refs 6, 7 and 23. Aggregation is described as a linear polymerisation process, where monomers can nucleate in solution, with rate constant k_n_ and reaction order n_c_ in monomer, in a process referred to as primary nucleation. These nuclei can then elongate by addition of monomers to the end of the forming fibrils with rate constant k_+_. Fibrils may catalyse the formation of further growth competent fibril ends either by surface catalysed secondary nucleation (with rate constant k_2_ and reaction order n_2_ in monomer) or by fragmentation (with rate constant k-, independent of the monomer concentration). A scheme of these processes is shown in Fig. 3 and the contributions of each process to the rate equations are shown in Figure S3. The different models considered here are distinguished mainly by the presence and nature of their secondary mechanism of formation of new fibrils.

Four models were considered here, the detailed equations can be found in the relevant references, and the composite steps are marked in Fig. 3.

- Model 1: Simple nucleated polymerisation, including only primary nucleation and elongation of fibrils^30^.
- Model 2: Like model 1, with the addition of fragmentation as a secondary source of new fibrils^23^,^31^.
- Model 3: Like model 1, with the addition of surface catalysed secondary nucleation as a source of new fibrils. This model was found to describe the aggregation of Aβ42 wild type^6^.
- Model 4: Like model 3, but treating the surface catalysed secondary nucleation process in more detail, as a 2 step mechanism, which opens the possibility of saturation of this step at high monomer concentrations^7^.

The fitting of these models to the data was performed using the Amylofit interface (http://amylofit.ch.cam.ac.uk/)^25^. The four models described above were tested and the plots for all fits are shown in Figure S1. Each model was tested with reaction orders fixed to the values (n_c_ = n_2_ = 2), as obtained in the case of Aβ42^6^ (Figs S1 and S2, left column) and also with n_c_ and n_2_ as fitted parameters (Figs S1 and S2, right column).

## Additional Information

**How to cite this article**: Meisl, G. *et al.* Quantitative analysis of intrinsic and extrinsic factors in the aggregation mechanism of Alzheimer-associated Aβ-peptide. *Sci. Rep.* **6**, 18728; doi: 10.1038/srep18728 (2016).

## Supplementary Material

Supplementary Information
