# Supplementary material for: Quantitative analysis of intrinsic and extrinsic factors in the aggregation mechanism of Alzheimer-associated Aβ-peptide
Source: Sci Rep. 2016 Jan 13;6:18728. doi: 10.1038/srep18728 (PMC4725935; doi:10.1038/srep18728)
Supplement: Supplementary Information [file srep18728-s1.doc]

**Supplementary Information for**

**Quantitative analysis of intrinsic and extrinsic factors in the aggregation mechanism of Alzheimer-associated Aβ-peptide**

Georg Meisl^1^, Xiaoting Yang^2^, Birgitta Frohm^2^, Tuomas PJ Knowles^1^, Sara Linse^2^

^1^ Department of Chemistry, University of Cambridge, Lensfield Rd, Cambridge, CB21EW, UK.

^2^Department of Biochemistry and Structural Biology, Lund University, Lund, 22100, Sweden.


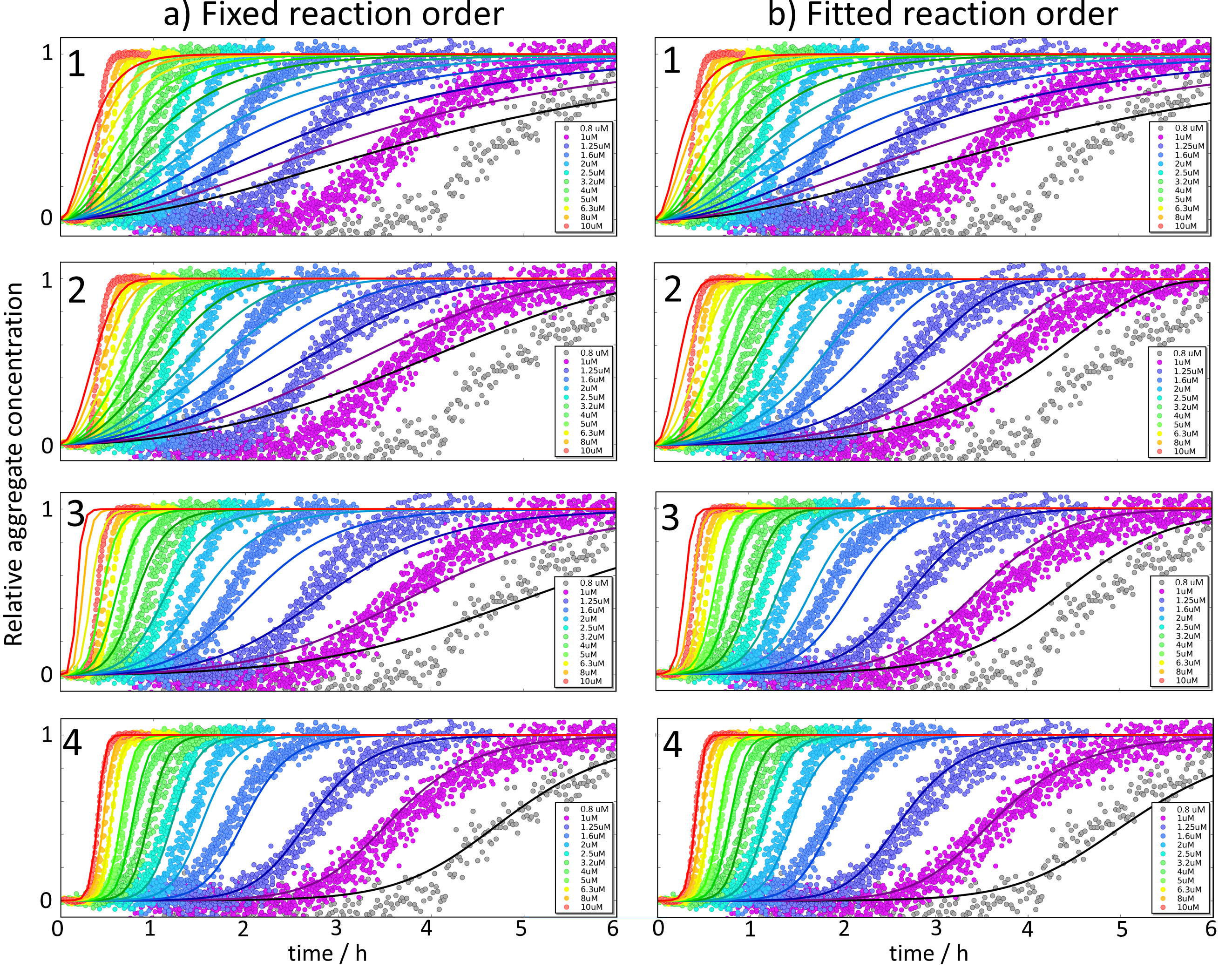


**Figure S1.** Normalized aggregation time courses for Aβ42-A2V starting from 0.8 (grey), 1.0 (purple), 1.25 (marine), 1.6 (blue), 2.0 (light blue), 2.5 (cyan), 3.2 (green), 4 (green), 5 (green), 6.3 (yellow), 8 (orange) and 10 µM (red) Aβ42-A2V monomer in 20 mM NaP, 0.2 mM EDTA. 0.02% NaN_3_, pH 8.0. Each colour shows four technical replicates at each concentration. Four models were fitted to the data and include the following microscopic steps: **1)** Primary nucleation and elongation. **2)** Primary nucleation, fragmentation and elongation. **3)** Primary nucleation, secondary nucleation and elongation. **4)** Primary nucleation, multi-step secondary nucleation and elongation. To the left (a) are shown fits with fixed reaction orders n_c_ = n_2_ = 2, and to the right (b) fits with the reaction orders as free parameters. The total number of free parameters per model are 1 (k_n_k_+_; model 1a), 2 (k_n_k_+_ and n_c_; model 1b), 2 (k_n_k_+_ and k_+_k-; model 2a), 3 (k_n_k_+_, k_+_k-, and n_c_; model 2b), 2 (k_n_k_+_ and k_2_k_+_; model 3a), 4 (k_n_k_+_, k_n_k_+_, n_c_ and n_2_; model 3b model 3b), 3 (k_n_k_+_, k_2_k_+_, and √K_M_; model 4a), and 5 (k_n_k_+_, k_2_k_+_, n_c_, n_2_ and √K_M_; model 4b).

**
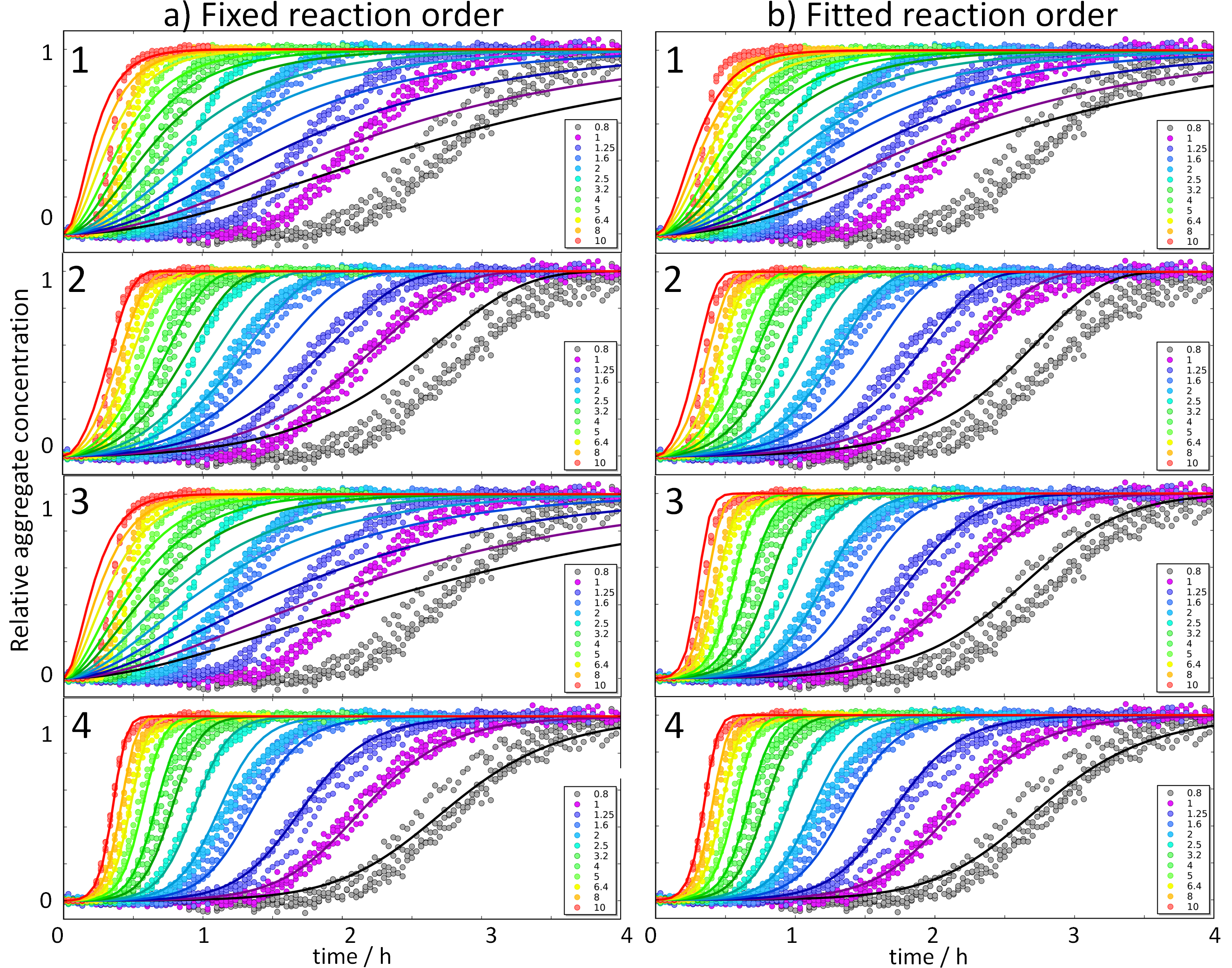
Figure S2.** Normalized aggregation time courses for Aβ42 at pH 7.4 starting from 0.8 (grey), 1.0 (purple), 1.25 (marine), 1.6 (blue), 2.0 (light blue), 2.5 (cyan), 3.2 (green), 4 (green), 5 (green), 6.3 (yellow), 8 (orange) and 10 µM (red) monomer in 20 mM NaP, 0.2 mM EDTA. 0.02% NaN_3_, pH 7.4. Each colour shows four technical replicates at each concentration. Four models were fitted to the data and include the following microscopic steps: **1)** Primary nucleation and elongation. **2)** Primary nucleation, fragmentation and elongation. **3)** Primary nucleation, secondary nucleation and elongation. **4)** Primary nucleation, multi-step secondary nucleation and elongation. To the left (a) are shown fits with fixed reaction orders n_c_ = n_2_ = 2, and to the right (b) fits with the reaction orders as free parameters. The total number of free parameters per model are 1 (k_n_k_+_; model 1a), 2 (k_n_k_+_ and n_c_; model 1b), 2 (k_n_k_+_ and k_+_k-; model 2a), 3 (k_n_k_+_, k_+_k-, and n_c_; model 2b), 2 (k_n_k_+_ and k_2_k_+_; model 3a), 4 (k_n_k_+_, k_n_k_+_, n_c_ and n_2_; model 3b model 3b), 3 (k_n_k_+_, k_2_k_+_, and √K_M_; model 4a), and 5 (k_n_k_+_, k_2_k_+_, n_c_, n_2_ and √K_M_; model 4b).

**Figure S3.** Mechanisms of aggregation (adapted from reference 7). A) The processes that make up the reaction network of aggregation, with their contributions to the increase in fibril number, P, and fibril mass, M, as well as the dependence of their kinetics on the monomer and fibril concentrations. B) The secondary nucleation mechanism in further detail, showing the monomer dependent first attachment step, followed by the monomer independent formation/detachment step. Above a certain monomer concentration, determined by the Michaelis constant K_M_=(k_2_+k_b_)/k_f_, this system becomes saturated. At a monomer concentration equal to √K_M_, the secondary nucleation process is half saturated. This is the model that was found to give the best agreement with the aggregation data.

**Kinetic analyses of seeded data**

The performance of seeded experiments serves two purposes. First, it allows one to test the predictions made by the model fitted to unseeded data and second, it can be used to obtain an estimate of the individual rate constants of the microscopic processes: Due to the nature of an unseeded aggregation reaction, only a combination of the rate constants can be obtained from such data, namely k_n_k_+_, k_2_k_+_ and k_+_k-. An estimate of the elongation rate would allow one to obtain the individual rate constants from these values. Such an estimate can be obtained by considering seeded aggregation data, however, the initial conditions of such a reaction, namely the seed number and mass need to be known in order to extract k+. Whilst the seed mass is easily determined by a measurement of the monomer concentration after dissociation of the fibrils, the seed number is more difficult to obtain and usually only approximate. In our case the average length of the seeds (~7000 monomers to within a factor of 3) was estimated from the TEM data. Using this value and the value of the combined rate constants obtained from the fits of unseeded data, the seeded data can be fitted globally with only a single free parameter, the elongation rate k_+_. These fits are shown in Fig. 4E and Fig. 5E in the main text. In this case the rate of elongation was found to be k_+_ = 1.8.10^7^ M^-1^s^-1^, which is within the error of the value found for the wild type (k_+_ = 3.10^6^ M^-1^s^-1^, ref 6). So whilst seeded experiments are a stringent test for the validity of the proposed model under varied conditions, it should be emphasized, that the inaccuracy of the measurement of the seed length will directly translate to the inaccuracy in the calculated individual rate constants, k+, k_n_ and k_2_. Hence, whereas the combined rate constants can be determined to good accuracy, the individual values obtained in this way should be treated only as estimates. We therefore focus on a comparison of k_n_/k_2_ and k_2_k_+_, which can be determined accurately.
